# Energy Expenditure Evaluation in Humans and Non-Human Primates by SenseWear Armband. Validation of Energy Expenditure Evaluation by SenseWear Armband by Direct Comparison with Indirect Calorimetry

**DOI:** 10.1371/journal.pone.0073651

**Published:** 2013-09-19

**Authors:** Francesca Casiraghi, Raweewan Lertwattanarak, Livio Luzi, Alberto O. Chavez, Alberto M. Davalli, Terry Naegelin, Anthony G. Comuzzie, Patricia Frost, Nicolas Musi, Franco Folli

**Affiliations:** 1 Department of Medicine/Division of Diabetes, University of Texas Health Science Center at San Antonio, San Antonio, Texas, United States of America; 2 Department of Biomedical Sciences for Health, University of Milan, Milano, Italy; 3 Metabolism Research Center, I.R.C.C.S. Policlinico San Donato Hospital, Milano, Italy; 4 Texas Diabetes Institute, San Antonio, Texas, United States of America; 5 Department of Internal Medicine, Ospedale San Raffaele, Milano, Italy; 6 Southwest National Primate Research Center, San Antonio, Texas, United States of America; 7 Department of Genetics, Texas Biomedical Research Institute, San Antonio, Texas, United States of America; University of Tor Vergata, Italy

## Abstract

**Introduction:**

The purpose of this study was to compare and validate the use of SenseWear Armband (SWA) placed on the arm (SWA ARM) and on the back (SWA BACK) in healthy humans during resting and a cycle-ergometer exercise and to evaluate the SWA to estimate Resting Energy Expenditure (REE) and Total Energy Expenditure (TEE) in healthy baboons.

**Methods:**

We studied 26 (15F/11M) human subjects wearing SWA in two different anatomical sites (arm and back) during resting and a cycle-ergometer test and directly compared these results with indirect calorimetry evaluation (IC), performed at the same time. We then inserted the SWA in a metabolic jacket for baboons and evaluated the TEE and REE in free living condition for 6 days in 21 (8F/13M) non-human primates.

**Results:**

In humans we found a good correlation between SWA place on the ARM and on the BACK with IC during the resting experiment (1.1±0.3 SWAs, 1±0.2 IC kcal/min) and a slight underestimation in the SWAs data compared with IC during the cycle-ergometer exercise (5±1.9 SWA ARM, 4.5±1.5 SWA BACK and 5.4±2.1 IC kcal/min). In the non-human primate (baboons) experiment SWA estimated a TEE of 0.54±0.009 kcal/min during free living and a REE of 0.82±0.06 kcal/min.

**Conclusion:**

SWA, an extremely simple and inexpensive apparatus, provides quite accurate measurements of energy expenditure in humans and in baboons. Energy expenditure data obtained with SWA are highly correlated with the data obtained with “gold standard”, IC, in humans.

## Introduction

The precise evaluation of Energy Expenditure (EE) during free living conditions is important in order to prevent and manage the increasing sedentary lifestyle [1]. In fact, physical inactivity is the fourth leading cause of death worldwide and a critical determinant of many chronic pathological condition such as obesity, insulin resistance and type 2 diabetes mellitus (T2DM), among others [2], [3].

The gold standards for measuring Resting Energy Expenditure (REE) and Total Energy Expenditure (TEE) in free living conditions are Indirect Calorimetry (IC) and the Double Labeled Water (DLW) techniques, respectively. These techniques are quite elaborate, expensive and can be performed only in few selected centers around the World.

Questionnaire, pedometers and accelerometers were the first alternative methods used to have an accurate and reliable assessment of EE during physical activity [4]–[6].

The next generation of these devices is represented by multisensory activity and lifestyle monitors that provide an estimate of REE and TEE and improve the measurement of the physical activity by using different algorithms.

SenseWear Armband (SWA; BodyMedia, Inc., Pittsburgh, PA) is a multisensory activity monitor. The device is typically worn on the upper right arm and provides estimation of TEE during free living based on a biaxial accelerometer, the galvanic skin response and the body heat loss.

SWA has been validated in different populations including adults [7]–[11] , children [12]–[14] and patients affected by different diseases [15]–[19], both during resting [9], [11], [20], [21] as well of different intensity physical activities [22]–[27].

The non-human primate is a valuable animal model in biomedical research, thanks to its close phylogenetic proximity to humans. We have previously demonstrated that baboons display most if not all the critical pathophysiological and molecular alterations that are typically seen in obesity, insulin resistance, and T2DM in humans [28]–[34].

In this study, we assessed in humans and non-human primates, baboons, the REE and TEE by using a new activity monitor called SWA. In humans, SWA is generally placed on the arm, but this body site cannot be used in baboons as they would remove it, in few minutes.

To overcome this problem, we decided to: 1) test and validate the reliability of SWA placed in two locations of the human body, the arm and the back (latissimus dorsi muscle); 2) compare the results obtained with SWA placed on the arm or the back with indirect calorimetry during resting and during a cycle ergometer test in humans; 3) test the SWA on baboons, in the back, after mounting it in a special jacket that would prevent the animals to remove it.

Therefore, the objectives of this study were three: 1) to compare SWA EE measurements after placing the device in two different areas of the human body, i.e. the right arm and the latissimus dorsi muscle, with EE measured by IC, in resting conditions; 2) to compare SWA EE measurements in the same body areas and EE measured by IC, during intense physical activity 3) after having established the correlation between SWA EE measurements obtained from arm and trunk in humans in resting condition and during intense physical activity, to evaluate REE and TEE in free living condition in baboons over a period of 6 days with the SWA placed on the latissimus dorsi muscle in a special jacket.

## Materials and Methods

SWA is a wireless multisensory activity monitor; it is normally worn on the upper right arm over the triceps muscle, halfway between the acromion and olecranon. The SWA collects and processes a variety of physiological data through multiple sensors I): a two-axis accelerometer; II): heat flux sensor; III): skin temperature sensor; IV): near-body ambient temperature sensor; V): galvanic skin response sensor) that can then be uploaded and analyzed using a computer software called InnerView Research Software (InnerView Research Software 6.1 ; BodyMedia Inc, Pittsburgh, PA, USA).

### Human Subjects

Twenty six healthy participants (n = 15 females and n = 11 males) were enrolled in the study protocol. At the beginning of the study, each subject underwent a general history evaluation and physical examination and provided written informed consent. The study was approved by the University of Texas Health Science Center at San Antonio's Institutional Review Board. Measurements were taken in the morning after an overnight fast and free of structured physical exercise for at least 48 hours.

Upon arrival to the laboratory, the subject's weight and height were measured. BMI was calculated as weight (kg)/height (m^2^).

Dual energy X-ray Absorptiometry (DXA) was also performed in all subjects.

Before starting the test at each subject was asked additional personal data, such as date of birth, right or left handed, and smoking history to complete the personalized setting of the SWA using the InnerView Research Software 6.1 (BodyMedia Inc, Pittsburgh, PA, USA).

### Evaluation of Resting Energy Expenditure in Humans

In these studies, we evaluated 22 subjects (n = 13 females, n = 9 males). Their anthropometric characteristics are reported in Table 1.

**Table 1:** Descriptive characteristic of human subjects (n = 26).

Subjects	ALL (N = 26, F = 15, M = 11)			RESTING (N = 22,F = 13,M = 9)			EXERCISE (N = 18,F = 11,M = 7)		
	mean ± SD			mean ± SD			mean ± SD		
**AGE (years)**	46.1	±	19.4	44.5	±	19.6	48.6	±	21.0
**HEIGHT (m)**	1.7	±	0.1	1.7	±	0.1	1.7	±	0.1
**WEIGHT (kg)**	73.5	±	17.8	74.1	±	18.3	68.2	±	13.9
**BMI (kg/m^2^)**	26.2	±	4.4	26.0	±	4.7	24.6	±	2.6
**FFM (kg)**	47.4	±	13.2	47.7	±	13.4	44.9	±	12.3
**FM (kg)**	23.6	±	8.2	23.9	±	8.2	20.9	±	6.3
**% FAT**	32.3	±	7.8	32.5	±	7.3	31.2	±	8.3

The purpose of this test was to evaluate the reliability of the EE data obtained by the SWA placed in 2 different areas of the body on the arm (SWA ARM) and on the back (SWA BACK) and comparing these data provided by IC during a 30 min resting period.

During this experiment, we allowed our subjects to rest on a bed for 1 hour lying down in a comfortable position, before starting the IC procedure. This procedure was performed using a plastic hood ventilation system (Vmax Encore, Viasys Healthcare, Yorba Linda, CA) for 30 min while the patient was lying down supine.

In order to allow acclimatization, both SWA ARM and SWA BACK (SWAs) were placed before the subject was lying down on the bed. One was placed on the upper right arm, as recommended by the manufacturer, and the second one around the waist in the lumbar zone on the latissimus dorsi muscle.

### Evaluation of Energy Expenditure during Exercise in Humans

In these studies, we evaluated 18 subjects (n = 11 females, n = 7 males). Their anthropometric characteristics are reported in Table 1.

The second experiment was carried out to evaluate the EE during a cycle-ergometer test, comparing the EE data estimated from the SWAs with EE data obtained with the IC employing the SensorMedics Vmax 29 apparatus (SensorMedics Inc, Yorba Linda, CA, USA).

For each subject we determined the Oxygen Consumption (VO_2_) using a incremental exercise test on a cycle-ergometer Ergometrics 800 (SensorMedics Inc, Yorba Linda, CA, USA) with continuous IC measurement through the SensorMedics Vmax 29 apparatus. At the same time the subjects were also wearing an SWA on the upper right arm and another SWA around the waist, on the lumbar muscles.

We divided the test into three different parts. The first minute was defined as Baseline (B) where the subject sat on the cycle-ergometer without riding. The second part was defined the Warm Up (W), where the subject started to ride the bike for 2 minutes at 40 rpm (speed) at 40 watts (intensity). After the end of the 3rd minute, the last period defined Exercise (E) started, where the speed rose up to 60 rpm and the intensity increased every minute (7 Watts/min) until exhaustion, which was determined when the subjects failed to maintain 60 rpm.

Before each test, the mouthpiece was given to the subjects while they were sitting on the cycle-ergometer, and the SensorMedics Vmax 29 system calibration was performed. The EE data were collected at one minute intervals.

### Non-Human Primates (Baboons) Subjects

Twenty one baboons *(Papio hamadryas),* 8 females and 13 males were involved in this study.

In order to get anthropometric measurement in the baboons, they were sedated and then weight (kg), height (m) and waist circumference (m) were measured, BMI was calculated as weight (kg)/stature (m^2^); all the anthropometric characteristics are reported in Table 2.

**Table 2:** Descriptive characteristics of the baboons (n = 21).

Primates	Total n = 21			Female n = 8			Male n = 13		
	mean ± SD			mean ± SD			mean ± SD		
**AGE (years)**	12.2	±	3.8	15.1	±	3.2	10.5	±	2.9
**HEIGHT (m)**	1.0	±	0.1	0.9	±	0.1	1.1	±	0.1
**WEIGHT (kg)**	27.0	±	7.4	18.8	±	0.8	32.1	±	4.2
**WAIST CIRCUMFERENCE (m)**	0.6	±	0.1	0.5	±	0.1	0.6	±	0.1
**BMI (kg/m^2^)**	24.8	±	4.4	21.5	±	2.4	26.8	±	4.1

### Evaluation of Free Living Energy Expenditure in Non-Human Primates

Since it is not possible to keep the SWA on the arm of the baboon because of their innate curiosity and tendency to remove the device, we placed the metabolic holter in a specially designed “metabolic jacket”, modified from the one used in the tether system of the baboon that has a slit in the back allowing the placement of the SWA to be in contact with the skin of the baboon's lumbar area on the latissimus dorsi muscle [35].

We tested the SWA placed in a special jacket in 21 baboons (n = 8 females, n = 13 males).

In the first day, the animals were sedated, weight (kg), height (m) and waist circumference (m) were measured and all the needed data inserted in the InnerView Research Software in order to set the SWA before placing it on the jacket.

All animals were housed in a single cage, for 1 week with ad libitum access to water and food (500 g of chow daily plus enrichment such as grains, various kind of fruits and vegetables, peanut butter, dry fruit, honey, cereal, and frozen yogurt).The standard chow contained 57.7% carbohydrates, 15.3% protein, and 4.7% fat (Monkey Diet 15%, Purina 5LE0; TestDiet; Richmond, IN). Enrichment games were constituted by videos and balls.

Baboons were sedated with ketamine to allow placement of the jacket with the embedded activity monitor. Animals were observed twice daily for any clinical or behavioral abnormalities which includes pain or discomfort. Each animal assigned to the study was acclimated to the jacket. Only animals that would accommodate the jackets were selected.

After placement of the metabolic jacket, they were returned to their cage and observed until recovered. The animals were also checked for potential signs of skin irritation produced by the jacket. There were no signs of pain or discomfort produced by the jacket throughout the study period.

After 6 days the animal were sedated again and the jacket with the SWA removed, and the data downloaded.

We evaluated also a REE on 30 min period in which the baboon was not moving and the light was off, and the percentage of contact of SWA with the body surface was 100%.

### Ethics Statement

Experimental protocols were approved by the Institutional Animal Care and Use Committee (IACUC) of the Texas Biomedical Research Institute and the University of Texas Health Science Center San Antonio and conformed to the current guidelines of the National Institutes of Health for the care and use of laboratory animals. All experiments carried on Human Subjects were approved by the University of Texas Health Science Center San Antonio Institutional Review Board (IRB).

### Statistical Analysis

Statistical analysis was performed with GraphPad 5.01 (GraphPad Software, Inc.).

The Bland-Altman bias plots were used to assess the agreement between the IC measurement and the SWA estimation (placed in the upper right arm and around the waist in the lumbar zone) during resting and activity. The limit of agreement (LOA) involved the mean difference between the two measurement tools ±1.96 SD of the differences.

Pearson's correlations were also used to analyze the correlation between the EE data provided by the SWA, placed on the arm or the back, and IC. Statistical significance was defined at p<0.05 and data are presented as mean ± standard deviation (SD) or mean ± standard error (SE).

## Results

**Figure 1:**
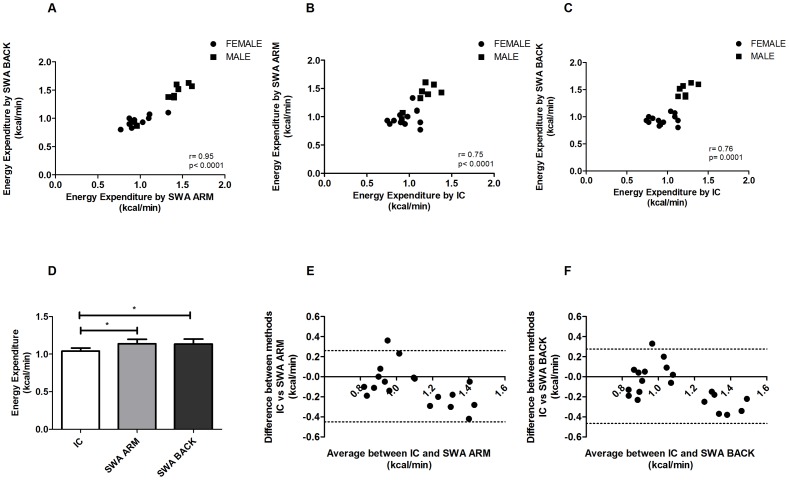
Human Resting. In figure 1 (A) Pearson's correlations between the EE estimate by SWA ARM and BACK in all subjects, in (B) correlation between SWA ARM and IC and (C) correlation between SWA BACK and IC. In panel D EE data divided by IC, SWA ARM and SWA BACK (mean ± SEM). Bland-Altman bias plot between SWA estimate in the ARM (E) and in the BACK (F) compared with IC (IC) measurements of EE during 30 min of resting.

In the first part of the study (resting), we compared the data obtained from SWAs and the IC, in healthy human subjects. The Pearson's correlation between the energy expenditure recorded by SWA ARM and the SWA BACK was extremely high, r = 0.95, p<0.0001 (Fig. 1A); there were also very high correlations between energy expenditures measured by SWA ARM and IC, r = 0.75, p<0.0001, and between SWA BACK and IC, r = 0.76, p = 0.0001 (Fig. 1B–C). Values for the SWA ARM and SWA BACK were highly super imposable, 1.1±0.3 kcal/min and data provided by the IC were 1±0.2 kcal/min with an apparent slight overestimation of the SWA as compared with the IC (Fig. 1D).

The Bland-Altman plot also demonstrated the good agreement between the two measurements in both SWA ARM as well as SWA BACK as compared with IC (Fig. 1E–F).

For the second part of our study (exercise), the EE values estimated by SWAs appeared to be slightly underestimated as compared with IC data during VO_2_.

**Figure 2:**
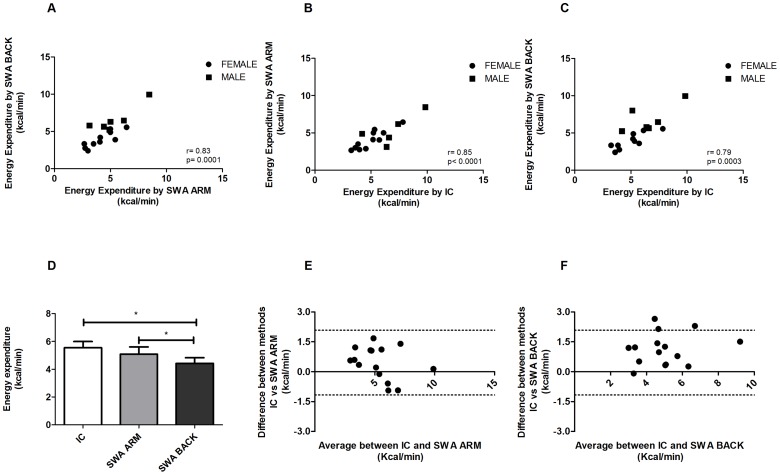
Human Exercise. Pearson's correlation between SWA estimate in the ARM and in the BACK (A) with VO_2_ measurement of EE during an incremental exercise on cycloergometer, correlation between SWA ARM and IC and (C) correlation between SWA BACK and IC. (D) EE data divided by IC, SWA ARM and SWA BACK (mean ± SEM). Bland-Altman bias plot between SenseWear Armband (SWA) estimate for the ARM (E) and for the BACK (F) and IC measurement of EE during an incremental exercise on cycloergometer.

The Pearson's correlation between the EE measured by SWA ARM and the EE measured by SWA BACK was very high, r = 0.83, p = 0.0001 (Fig. 2A); there were also highly significant correlations between SWA ARM and the VO_2_, r = 0.85, p<0.0001 and between SWA BACK and VO_2_, r = 0.79, p = 0.0003 (Fig. 2 B–C).

EE values recorded by SWA ARM were 5±1.9 kcal/min, EE values recorded by SWA BACK were 4.5±1.5 kcal/min and the values obtained by IC during exercise were 5.4±2.1 kcal/min. Data obtained by SWA BACK were statistically different compared with VO_2_ and SWA ARM (Fig. 2D).

The Bland-Altman plot again demonstrated a good agreement between the two measurements provided by SWA in both places, in the ARM and in the BACK, as compared with the results obtained by the IC during the exercise period (Fig 2E–F).

In the third part of the study we compared the data, provided by SWA placed in the metabolic jacket, during resting and during free living condition in baboons.

During the resting part the mean EE was 0.537±0.009 kcal/min for all the animals, 0.625±0.005 kcal/min for males and 0.367±0.007 kcal/min in females.

**Figure 3:**
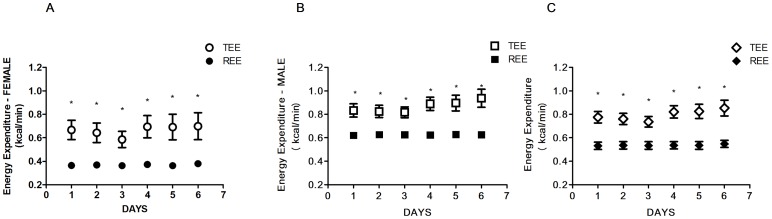
Baboon Activity and Resting. Plots of mean Energy Expenditure (kcal/min) during resting and activity in female (A), male (B) and in all the baboons (C).

In the TEE the overall mean was 0.82±0.06 kcal/min, 0.89±0.06 kcal/min for male, and 0.68±0.07 kcal/min for female (Fig 3 A–B–C).

We also compared the results obtained in the resting experiment in humans with the data provided by SWA placed in the metabolic jacket in baboons.

**Figure 4:**
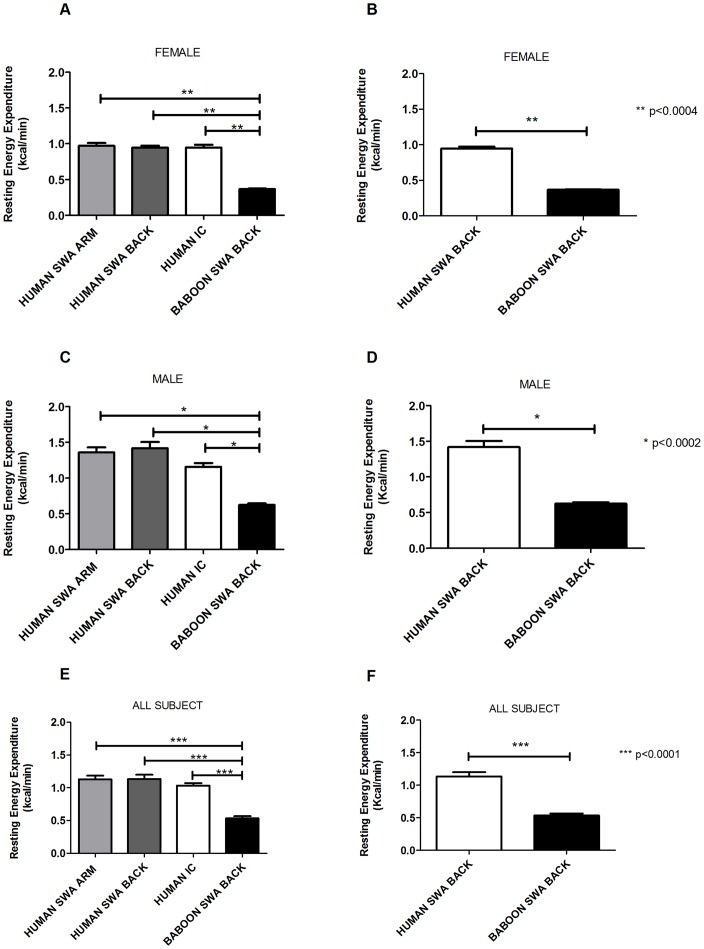
Comparison between humans and baboons. Comparison of EE at resting between humans and baboons, in the female groups (A) in all the locations placed and (B) only on the back , in the male group (C) in all the locations placed and (D) only on the back ; in all the subjects (E) in all the locations placed and (F) only on the back.

There was a statistical difference between human and baboons, suggesting a lower EE in baboons as compared to humans, in females p<0.0004 (Fig 4A–B), male p<0.002 (Fig. 4 C–D) and in all subjects (Fig. 4 E–F) p<0.0001.

## Discussion

A large variety of studies have been performed to test the reliability of the SWA by estimating EE during resting and physical activity and comparing the results with DLW [4], [7], [8], [36] , IC [9]–[14], [16]–[19], [21]–[23], [26], accelerometers and pedometers [37]; in controlled situation and in free living conditions [7], [10].

In this study we tested the reliability of the SWA by estimating EE during resting and during a cycle-ergometer test and compared the results obtained by wearing the SWA in two different areas of the body. We also explored the feasibility to register the EE in baboons placing SWA in a special metabolic jacket for 6 days in free living condition.

In the first part of the study we tested the accuracy of the SWA placed in 2 different muscular districts in the body, finding an extremely high correlation between the two different locations, i.e. arm and back, both in resting conditions as well as during exercise.

We also found a slight underestimation in data provided by SWA compared with IC and very good linear correlations between the SWAs and the IC data, similarly to what previously shown by other investigators [9], [11]. Papazoglou et al. [19] showed in 25 lean and overweight subjects with a BMI 25.3±3.2 kg/m^2^ a Bland-Altman plot for the IC measure sand SWA estimates with a high correlation (r = 0.96,p<0.001) and a very good agreement in the measurement of REE with the two methods. Heiermann et al [20] compared REE provided by IC and SWA in older adults, and found an overestimation by 12–14% in the data provided by SWA in morning and night recording respectively.

In the second part of our study we tested the reliability of the SWA in providing a correct measure of EE during a cycle-ergometer test until exhaustion. Our SWA data showed a small underestimation of EE as compared with the IC, but again as in the resting portion of the study, overall we demonstrated a very highly significant correlation between the data obtained with the SWA and IC. Our data are consistent with previously published studies, demonstrating a slight overestimation/underestimation of EE (around ∼10%) with SWA as compared with IC, but an excellent overall correlation between the two methods [17], [22]–[25], [27].

Fruin et al. [9] showed EE derived by cycling exercise was substantially lower than that from the IC and poorly correlated (r = 0.11, p<0.73).

SWA seemed to provide accurate and reliable estimations of REE in patients affected by cancer, Parkinson's disease, Chronic kidney disease and COPD [15], [16], [18], [38]. The SWA has also been utilized to monitor EE in obese patients after a bariatric surgery [39].

Our data are in agreement with some and somehow at variance with some of the previously cited studies, showing that SWA can be used successfully in humans and, for the first time that it can be employed also on the latissimus dorsi both in resting condition as well as during intense exercise.

This latest finding can have multiple applications, in different sports where arms are particularly stressed and the performance can be compromised if the SWA is placed on the arm, or in clinical studies when the SWA cannot be places on the triceps, such as for example in intensive care units, where monitors and infusion lines are placed on the arms.

In this clinical context, the measurement of the basal metabolic rate can be also important, for example, to provide optimal nutritional support in critical patients.

The last part of our study was focused on the adaptation of the SWA in the metabolic jacket for the baboons and measuring energy expenditure during 6 days of free living condition.

We believe it is interesting to show that this device can be used also in non-humans primate and SWA is able to detect differences in EE during resting and physical activity, also if the EE is very low due to small space (cage) where baboons were placed.

There are very few studies in literature about the daily energy expenditure of baboons.

Rosetta and collaborators measured with DLW, the total EE and compared the variations between two different periods of life, early lactation and after the resumption of sexual cycling, in 8 female baboons (*Papio Anubis*); 24 hours total EE was determined over a 4-day period; the average TEE was 3.49 MJ/d in the first period and 3.48 MJ/d in the second [40].

Leonard and collaborators examined the variations in metabolic requirements among extant primate species based , *Papio Anubis,* the Resting Metabolic Rate (RMR) was 956 kcal/d for male and 520 kcal/d for female; the TEE was 1281kcal/d for male and 699 kcal/d for female which are in agreement with our estimated EE employing the SWA [41]. Table 3 has been included to facilitate the comparison between the present study and the two previously published studies [40], [41].

**Table 3:** Comparison of Total Energy Expenditure (TEE) data (kcal/day) and Resting Energy Expenditure Data (REE) data (kcal/day) between three different studies in non-human primates.

	TEE (kcal/day)		REE (kcal/day)		Method
	Male	Female	Male	Female	
**Present study**	1267	936	893	533	SenseWear Armband (SWA)
**Rosetta et Al. (40)**	N/A	833	N/A	N/A	Doubly Labeled Water (DLW)
**Leonard et Al. (41)**	1281	699	956	520	Indirect Calorimetry + Estimation by Equations (Kleiber and Leonard)

N/A  =  Data Not Available.

Our study on human subjects has some limitations. We tested the SWAs in a medium size group of subject with different anthropometric characteristic in age and BMI and assumed that the IC measurements were stable and accurate in both measurements (resting and exercise).

However the study demonstrates that SWA can give highly reliable estimates of REE and slightly less, although still quite accurate, estimation on EE during a cycle-ergometer exercise. The results suggest that SWA could be used in large human population metabolic/exercise/pharmacological studies where IC would be not feasible to measure energy expenditure/metabolic rate because of its costly and laborious methodology, and the latissimus dorsi muscle can be also used as an alternative area to place the SWA.

Futures studies will help to confirm if SWA can be used to correctly estimate the TEE in healthy subjects and in subjects affected by different diseases, which aren't able to wear the armband in the upper right arm, but will wear the SWA on the back, in contact with the latissimus dorsi.

In conclusion, these studies demonstrate that the SWA is a reliable and simple method to estimate REE and EE worn on the right arm and on the lumbar district in humans, and to estimate TEE and REE in non-human primate, baboons, by placing it in the “metabolic jacket”.
